# GPS-Free Localization Algorithm for Wireless Sensor Networks

**DOI:** 10.3390/s100605899

**Published:** 2010-06-09

**Authors:** Lei Wang, Qingzheng Xu

**Affiliations:** 1 School of Computer Science and Engineering, Xi’an University of Technology, Xi’an 710048, China; E-Mail: leiwang@xaut.edu.cn; 2 Xi’an Communication Institute, Xi’an 710106, China

**Keywords:** wireless sensor networks, localization, GPS-free, local coordinate system, global coordinate system, matrix transform

## Abstract

Localization is one of the most fundamental problems in wireless sensor networks, since the locations of the sensor nodes are critical to both network operations and most application level tasks. A GPS-free localization scheme for wireless sensor networks is presented in this paper. First, we develop a standardized clustering-based approach for the local coordinate system formation wherein a multiplication factor is introduced to regulate the number of master and slave nodes and the degree of connectivity among master nodes. Second, using homogeneous coordinates, we derive a transformation matrix between two Cartesian coordinate systems to efficiently merge them into a global coordinate system and effectively overcome the flip ambiguity problem. The algorithm operates asynchronously without a centralized controller; and does not require that the location of the sensors be known *a priori*. A set of parameter-setting guidelines for the proposed algorithm is derived based on a probability model and the energy requirements are also investigated. A simulation analysis on a specific numerical example is conducted to validate the mathematical analytical results. We also compare the performance of the proposed algorithm under a variety multiplication factor, node density and node communication radius scenario. Experiments show that our algorithm outperforms existing mechanisms in terms of accuracy and convergence time.

## 1. Introduction

Technological advances in the areas of low energy cost wireless communication embedded computing, sensor and integrated circuits make it possible to implement large scale networks with hundreds and even thousands of very small, low-cost, battery-powered, and wirelessly connected sensor and actuator nodes. Wireless sensor networks (WSNs) can work unattended for long periods, and find a very wide range of applications in the fields of environmental monitoring, forest fireproofing, biology habitat monitoring and control, intelligent agriculture, intelligent architecture and houses, defending military targets, preventing terror attacks, individual health monitoring, *etc.* [1].

Imagine a network of sensors sprinkled across a large building or an area such as a forest or battlefield [2]. Typical tasks for such networks are to send a message to a node at a given location, even without knowing how many nodes there are or how to reach them, to retrieve sensor data (e.g., sound, light, radiation, temperature or humidity levels) from nodes in a given region, and to use the sensor nodes to track nearby events, such as vehicles moving through the sensor field. Most of these tasks require knowing the positions of the nodes or at least their relative positions. For example, for a vehicle-tracking application, the sensor nodes would determine the positions of the tracked vehicles relative to their own positions.

With a network of thousands of nodes, it is unlikely that the position of each node can be precisely predetermined. Although GPS based localization schemes can be used to determine node locations within a few meters, the cost of GPS devices and the non-availability of GPS signals in confined environments prevent their use in large scale sensor networks.

The localization problem in wireless sensor networks is how to determine the location information of all or a subset of sensor nodes, given the measurements of pairwise spatial relationships between the nodes [3,4]. In the literature, WSNs localization methods can normally be categorized according to three different aspects: (i) the information requirements of the solution schemes: proximity-based localization, range-based localization, angle-based localization and probabilistic-based localization; (ii) the hardware requirements of the solution schemes: absolute localization and relative localization, and (iii) the type of network structure: static network and mobile network.

In this paper, we present a GPS-free localization scheme for node localization in WSNs called the Matrix transform-based Self Positioning Algorithm (MSPA), where the task is to use the distance information between nodes to determine the coordinates of static nodes in a 2D or 3D space. At the heart of the approach is the matrix transform technology and a totally distributed network structure is adopted. Similar to other relative localization algorithms, the coordinate establishment phase is split into two phases: the establishment of local coordinates at a subset of the nodes and the convergence of the individual coordinate systems to form a global coordinate system. Obviously, our approach is a range-based relative localization algorithm applied in a stationary sensor network.

In this paper we make several departures from previous research. In the first phase, our contribution for developing a standardized process of constructing local coordinate systems is to exploit a new parameter, the multiplication factor *α*, which can regulate the number of master and slave nodes in sensor networks and the degree of connectivity between master nodes. In the second phase, we derive a transformation matrix between two local coordinate systems to efficiently merge them into a global coordinate system by means of homogeneous coordinates commonly used for transformations in computer graphics. Our approach can effectively overcome the potential flip ambiguity problem, taking into consideration the reflection transformation between two Cartesian coordinate systems. Another important issue in GPS-free localization algorithms is the fundamental performance impact of different parameters. In particular, a set of parameter-setting guidelines for the proposed algorithm is established by making use of a probability approach. A simulation analysis on a specific numerical example is conducted to validate the mathematical analytical results of the proposed parameter-setting guidelines.

The remainder of the paper is organized as follows. In the following section we give an overview of the related work. In Section 3, we provide a detailed description of our MSPA. Section 4 investigates the energy requirements and develops a probability model for parameter-setting guidelines. Section 5 shows some simulation results to characterize the performance of our algorithm. Finally, Section 6 concludes the paper.

## 2. Related Work

Self-localization capability is a highly desirable feature of wireless sensor networks. Sensing data are not useful unless the location from where the data are collected is known to the end users. For this reason, localization and positioning of sensor nodes is an important area that has attracted much research attention, and a range of algorithms have been reported in the literature for this purpose.

Most of the current literature on location discovery in WSNs assumes the availability of GPS receivers at some nodes or beacon nodes with known position [5–12]. Their locations are then used to determine the positions of other ordinary sensor nodes, which do not have GPS receivers. Having a GPS receiver at sensor nodes may not be feasible due to the limitations of satellite coverage or obstructions in the path of satellite signals or harsh climate conditions. All of the methods above are absolute localization ones because the ground truth position or global coordinate in a specific environment is acquired. Another kind of localization estimation is called relative localization, in which all devices in the network, regardless of their absolute coordinate knowledge, estimate the range between themselves and their neighboring devices. An absolute localization can be transformed into a relative localization—relative to a second reference point, that is. However, a second absolute localization is not always available [13].

The Self Positioning Algorithm (SPA), which is used in distributed mobile wireless networks without GPS receivers was proposed first by Capkun. In [14,15] every node establishes its local coordinate system by setting itself as the origin. Two other nodes are randomly chosen under the condition that the three nodes do not lie on the same line and can communicate with each other. Then, any other node can be localized if the distance to each of the three nodes can be estimated. After choosing the group of nodes having the highest density in the network as the localization reference coordinate system, the other local coordinate systems can be adjusted to build the global coordinate system by coordinate transformation. The disadvantage of SPA is that the communication cost and convergence time grow exponentially with the number of nodes since each node participates individually in the process of building and merging the local coordinate system.

To overcome the shortcomings of SPA, Cluster based SPA (CSPA) was proposed in [16]. In this method, the nodes are initialized with different roles: a small subset of the total number of nodes is selected to be master nodes and the others then become slave nodes. Every master node builds a local coordinate system with an algorithm similar to SPA, and two master nodes need two common slave nodes to merge their local coordinate systems. Compared to SPA, CSPA provides a considerable improvement by reducing communication overhead and convergence times and has been extended in the literature [17–20] to different applications. Unfortunately, the algorithm brings with it the flip ambiguity when merging local coordinate systems because two master nodes cannot be within the range of each other, the quadrilateral composed by two master nodes and two slave nodes is not a robust one [21,22].

Recently, the so called Backbone-based SPA (BSPA) was presented, in which some nodes in the network are first selected and then formed into a backbone network [23]. Because the local coordinate system is established only at the nodes in the backbone and then the backbone is a connected network, the method to adjust the directions of two overlap local coordinate systems and the approach to merge them proposed in [14,15] can be applied here directly, and the flip ambiguities can be avoided by robustness checking.

For more details of the principles and characteristics of other relative localization algorithms, we refer the reader to Chen’s good review [24]. Despite a significant number of relative localization approaches developed for WSNs localization, there are still many open issues in the area. First of all, all these relative location algorithms are based on topological relations between two sensor nodes in the merging phase, which is too complicated and expensive in terms of communication cost and convergence time. Another relevant research problem is analyzing and designing energy efficient layout schemes since sensors are battery-constrained devices. Obviously, this is a non-trivial task as it covers many unrelated tasks such as localization related measurements, communication among neighbors and estimating locations, among others. Through much research has studied the localization problem with the emphasis on algorithms, a more challenging research problem is that very few works focus on parameter selection criterion having direct impact on algorithm performance and field applications.

## 3. Matrix Transform Based SPA

### 3.1. Assumptions and Scheme Overview

In this paper, we investigate a GPS-free node localization algorithm for WSNs. It is assumed that distance measurements are already made hop-by-hop between neighboring sensor nodes. There are a number of technologies and techniques for performing the distance measurement. The simplest way is to measure the received signal strength and then apply a path loss model, such as the log path loss model [25] or the Walfisch-Ikegami model [26], to calculate the distance. The distance can also be determined based on the time-of-arrival measurements and the measurements of round-trip time-of-flight of a radio signal [27,28]. When both a radio signal and an ultrasound signal are employed, an extremely accurate time-of-flight estimate and hence distance estimate can be obtained [29]. We make the following main assumptions on our model:

- All sensors are stationary. So the network topology is fixed.
- There are no landmarks in the network. That is to say, no sensor has absolute location information.
- All sensors are homogeneous, with the same technical characteristics, and especially the same transmission range.
- All sensors have enough energy to accomplish a node localization algorithm.
- All sensors use omnidirectional antennae.
- All the wireless links between sensors are bidirectional.
- There are no base stations to coordinate or supervise activities among sensors. Hence, the sensors must make all decisions without reference to a centralized controller.

In a stationary wireless sensor network, as sensors are battery-constraint devices, network topology may change in two cases: some sensors will die away or go to sleep periodically, and some sensors will be resupplied to maintain the work efficiency and quality of the whole network. In general, we run our proposed MSPA when all sensor nodes are deployed in some area. After that, the location information of all sensor nodes will remain during all lifetime of sensor networks even some sensor nodes die due to energy exhaustion or go to sleep periodically to save their energy. In the latter case, we can use other absolute localization approaches to determine the coordinates of new sensor nodes because the former sensor nodes which know their global coordinate now can be considered as beacons. The simplest and the most widely used absolute localization approach is trilateration, which is suitable for our purposes because the number of new sensor nodes is trivial compared with hundreds and even thousands of former sensor nodes.

Similar to the other relative localization algorithms, the coordinate establishment phase of MSPA is split into two phases: the local coordinate establishment at master nodes and the convergence of the local coordinate systems to form a global coordinate system. The following sections describe the two phases in more detail.

### 3.2. Phase I: Construction of Local Coordinate Systems

Scalability and the need to conserve energy and reduce communication overhead led to the idea of organizing the sensors hierarchically, which can be accomplished by gathering collections of sensors into clusters [30]. Clustering sensors, which are the core ideas of CSPA and its improved algorithms, are advantageous because they: (i) conserve limited energy resources and improve energy efficiency, (ii) aggregate information from individual sensors and abstract the characteristics of network topology, (iii) provide scalability and robustness for the network.

In the following discussion, we assume that a number of sensors are deployed randomly over a geographical region with a given average density. After deployment, each sensor starts to decrement a random waiting timer. If the timer of node *i* expires, then the sensor *i* broadcasts a message M_1_ with a multiplication factor *α* ≥ 1 proclaiming that it is beginning a *master node*, a focal point of a new cluster. All nodes in the communication range of node *i* who receive this message become a *slave node*. We refer to some nodes, which hear from other master nodes as the *border nodes*. At the same time, all nodes hearing the message M_1_ also transmit messages M_2_ to their neighbor nodes announcing their existence and the distance between them. However, events may occur and cause a sensor to extend or stop its timer. For example, if a neighbor declares itself to be a master node, the sensor lengthens the timer. On the other hand, whenever the timer is greater than threshold value, the sensor cancels its own timer. The complete procedure of the clustering phase is outlined in Algorithm 1. After clustering phase, there are three different kinds of sensor: master nodes, slave nodes and border nodes.

**Table:** Algorithm 1

(1) Each sensor initializes a random waiting timer with a value *WT_i_*^(0)^ ∈ (0, *T_max_*) and initial status *S_i_* = none (*i* = 1, 2, 3, …)
(2) Decrease all random waiting timer *WT_i_*^(*k*)^
(3) Master node check:
*if* the random waiting timer expires, that is, *WT_i_*^(*k*)^ = 0
(a) *S_i_* = master node
(b) broadcast a message M_1_ with multiplication factor *α*
(c) delete the waiting timer
*end*
(4) Establish and update the neighbor identification:
*if* a sensor *S_j_* receives a message M_1_ at time step *k*
(a) *if S_j_* = slave node
*S_j_* = border node
*else*
*S_j_* = slave node
*end*
(b) transmit messages M_2_ to its neighbor nodes with the distance between node *i* and *j*
(c) *WT_j_*^(*k* + 1)^ = *α* × *WT_j_*^(*k*)^
(d) *if WT_j_*^(*k* + 1)^ > *T_max_*
delete the waiting timer
*end*
*end*
(5) Termination conditions check:
*if* the waiting timers of all sensors are deleted
algorithm is over
*else*
*k* = *k* + 1 and go to step (2)
*end*

Assume that the multiplication factor *α* = ∞, once a sensor receives a message M_1_ transmitted from master node *S_i_* at time step *k*, the random waiting timers of all neighbor nodes will be greater than the threshold value *T_max_*, and the neighboring sensors will not be master nodes any more. As a result, all master nodes are not connected in clustering sensor networks as in the literature [16]. In another special case of Algorithm 1 when the multiplication factor *α* = 1, the waiting timers of all nodes evidently will not change during one step under the same conditions. As time goes on, all nodes will inevitably become master nodes. There are, in essence, no concepts of slave nodes and border nodes in Algorithm 1 like in the literature [14,15]. Let us consider another case in which the multiplication factor *α* is related to the distance between two nodes *i* and *j*, that is *α = f* (*d_ij_*), and the more *d_ij_* is, the less *α* is. In this case, each master node has a greater chance of being deployed in the range of another master node. This procedure seems to coincide with that described in [23].

It is worth noting that more neighbor nodes can easily play the role of slave nodes during the early periods in Algorithm 1, while during the late ones, on the contrary, it is hard for neighbor nodes to become slave nodes. In order to avoid oscillation and keep the number of slave nodes and master nodes stable, one of the most viable solutions is the use of time factor in *α* to provide a little *α*(*t*) during the early stages and a large *α*(*t*) during the later stages.

The above implies that in the clustering phase, the multiplication factor *α* plays an important role in adjusting the number of master nodes and slave nodes and the degree of connectivity among master nodes and reducing communication overhead. It is reasonable to investigate the performance from the perspective of multiplication factor. For more details see Section 5.3.

**Figure 1.**
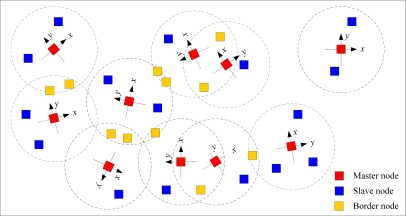
Wireless sensor network after the local coordinate system is established.

In this paper, we use triangulation to form the local coordinate system at each master node like in other relative localization algorithms [14–22]. For more details see Appendix A. Figure 1 depicts typical runs after the local coordinate system is established. The results show that all nodes in the sensor network are divided into three different kinds of sensor: master nodes, slave nodes and border nodes, the relationships between which have a significant impact on the localization. We can observe that some master nodes are at short ranges from each other, and show a master-slave relationship, while other master nodes are at a long distance, in which case the master-slave relationships are difficult to maintain, showing disjoint connections.

### 3.3. Phase II: Organizing a Global Coordinate System

Once the local coordinate systems have been constructed at the master nodes, all but one of the local coordinate systems needs to reorient their system in order for the network to converge to a single global coordinate system. In our opinion, merging two local coordinate systems is the process of one coordinate system coinciding with another using affine transformations including translation, reflection, rotation around any center, shearing and scaling [31]. In this paper, we only make use of translation, rotation and reflection transformations to adjust the coordinate systems in a 2-Dimensional WSNs, which preserves distances or angles and parallel relationships between two lines.

Homogeneous coordinates have a natural application to computer graphics; they form a basis for the projective geometry used extensively to project a three-dimensional scene onto a two dimensional image plane. Today, homogeneous coordinates are present in numerous computer graphics texts, computer-aided design/computer-aided manufacturing, robotics, surface modeling, computational projective geometry, and fixed point arithmetic [32]. An important, practical aspect of the homogeneous coordinate system is its unification of the translation, rotation and reflection transformations and operations of geometric objects. For example, in the 2-D Euclidean space, the homogeneous coordinates of a point *p*(*p_x_*, *p_y_*) is defined as (*p_x_*, *p_y_*, 1). Then, the most general affine transformation is:

(1) $$\left[p_{x}’\;p_{y}’\;1\right]\;=\;\left[p_{x}\;p_{y}\;1\right]\cdot T=\left[p_{x}\;p_{y}\;1\right]\cdot \left[\begin{array}{cc} T_{1} & T_{2}\\ T_{3} & T_{4} \end{array}\right]\;=\;\left[p_{x}\;p_{y}\;1\right]\cdot \left[\begin{array}{ccc} a & b & 0\\ c & d & 0\\ l & m & 1 \end{array}\right]$$

where **T** is a 3 by 3 matrix, namely a two-dimensional homogeneous coordinate transformation matrix (abbreviated as the transformation matrix). Considered from the functional point of view, the transformation matrix **T** can be divided into four sub-matrices, where $T_{1}=\left[\begin{array}{cc} a & b\\ c & d \end{array}\right]$ representing rotation, reflection, shearing and non-uniform scaling transformation, **T**_2_ = [*l m*] representing translation transformation, **T**_3_ representing projection transformation, and **T**_4_ representing uniform scaling transformation. In particular, $T_{3}=\left[\begin{array}{c} 0\\ 0 \end{array}\right]$ and **T**_4_ = [1] denote no projection and uniform scaling transformation, respectively.

Hence, according to the basic theory of computer graphics, the problem of organizing a local coordinate systems is converted into a mathematical optimization problem and solved by a linear matrix equation. Since there are six variables in the transformation matrix **T**, it is reasonable that we can solve Equation 1 using at most three group coordinates of the same sensor nodes in the two different coordinate systems, which are called border nodes.

**Figure 2.**
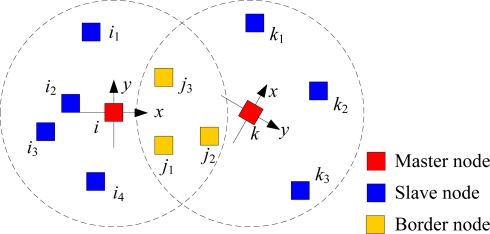
Merging local coordinate systems.

Let us consider two master nodes *i* and *k* as shown in Figure 2. In the following discussion, we assume, without loss of generality, that node *k* change its coordinates to the local coordinate system of node *i*. Suppose nodes *j*_1_, *j*_2_ and *j*_3_ are three border nodes, whose coordinates in different coordinate systems are (*j_x_*^1^, *j_y_*^1^), (*j_x_*^1′^, *j_y_*^1′^), (*j_x_*^2^, *j_y_*^2^), (*j_x_*^2′^, *j_y_*^2′^), (*j_x_*^3^, *j_y_*^3^) and (*j_x_*^3′^, *j_y_*^3′^), respectively. Thus, we can obtain homogeneous transformation equation as:

(2) $$\left[\begin{array}{ccc} j_{x}^{1\prime } & j_{y}^{1\prime } & 1\\ j_{x}^{2\prime } & j_{y}^{2\prime } & 1\\ j_{x}^{3\prime } & j_{y}^{3\prime } & 1 \end{array}\right]=\left[\begin{array}{ccc} j_{x}^{1} & j_{y}^{1} & 1\\ j_{x}^{2} & j_{y}^{2} & 1\\ j_{x}^{3} & j_{y}^{3} & 1 \end{array}\right]•\left[\begin{array}{ccc} a & b & 0\\ c & d & 0\\ l & m & 1 \end{array}\right]$$

Thus, the transformation matrix **T** is given by:

(3) $$T=\left[\begin{array}{ccc} a & b & 0\\ c & d & 0\\ l & m & 1 \end{array}\right]={\left[\begin{array}{ccc} j_{x}^{1} & j_{y}^{1} & 1\\ j_{x}^{2} & j_{y}^{2} & 1\\ j_{x}^{3} & j_{y}^{3} & 1 \end{array}\right]}^{-1}•\left[\begin{array}{ccc} j_{x}^{1\prime } & j_{y}^{1\prime } & 1\\ j_{x}^{2\prime } & j_{y}^{2\prime } & 1\\ j_{x}^{3\prime } & j_{y}^{3\prime } & 1 \end{array}\right]$$

where the necessary and sufficient conditions for invertibility of $\left[\begin{array}{ccc} j_{x}^{1} & j_{y}^{1} & 1\\ j_{x}^{2} & j_{y}^{2} & 1\\ j_{x}^{3} & j_{y}^{3} & 1 \end{array}\right]$ is that three border nodes do not lie in the same line.

### 3.4. More Discussion about the Transformation Matrix

As in the construction of the local coordinate systems in phase I, the transformation matrix approach in organizing a global coordinate system is a measure of general applicability. It covers all situations mentioned and is solved in other literature.

A special situation is found in the literature [14,15,23] when adjacent two master nodes can communicate with each other, that is *i* ∈ *K_k_*, *k* ∈ *K_i_*, the transformation matrix **T** can be determined only by another one border node *j*.

It is well known that the authors in [16] calculate the transformation matrix **T** using two border node *j*_1_ and *j*_2_. In fact, if we consider translation and rotation, the transformation matrix **T** can be simplified further to $\left[\begin{array}{ccc} a & b & 0\\ -b & a & 0\\ l & m & 1 \end{array}\right]$ because rotation is defined by [*p_x_*’ *p_y_*’ 1] = [*p_x_* *p_y_* 1]•**T**_1_, where $T_{1}=\left[\begin{array}{cc} \text{cos}(\theta ) & -\text{sin}(\theta )\\ \text{sin}(\theta ) & \text{cos}(\theta ) \end{array}\right]$ for a rotation of *θ* radians counter-clockwise about the origin. As in the general case discussed above, suppose nodes *j*_1_ and *j*_2_ are two border nodes, whose coordinates in different coordinate systems are (*j_x_*^1^, *j_y_*^1^), (*j_x_*^1′^, *j_y_*^1′^), (*j_x_*^2^, *j_y_*^2^) and (*j_x_*^2′^, *j_y_*^2′^), respectively. Thus, we can obtain homogeneous transformation equation as:

(4) $$\left[\begin{array}{ccc} j_{x}^{1\prime } & j_{y}^{1\prime } & 1\\ j_{x}^{2\prime } & j_{y}^{2\prime } & 1 \end{array}\right]=\left[\begin{array}{ccc} j_{x}^{1} & j_{y}^{1} & 1\\ j_{x}^{2} & j_{y}^{2} & 1 \end{array}\right]•\left[\begin{array}{ccc} a & b & 0\\ -b & a & 0\\ l & m & 1 \end{array}\right]$$

Therefore, we obtain:

(5) $$\left[\begin{array}{c} a\\ b \end{array}\right]={\left[\begin{array}{cc} j_{x}^{1}-j_{x}^{2} & -(j_{y}^{1}-j_{y}^{2})\\ j_{y}^{1}-j_{y}^{2} & j_{x}^{1}-j_{x}^{2} \end{array}\right]}^{-1}\left[\begin{array}{c} j_{x}^{1\prime }-j_{x}^{2\prime }\\ j_{y}^{1\prime }-j_{y}^{2\prime } \end{array}\right]$$

where the necessary and sufficient conditions for invertibility of $\left[\begin{array}{cc} j_{x}^{1}-j_{x}^{2} & -(j_{y}^{1}-j_{y}^{2})\\ j_{y}^{1}-j_{y}^{2} & j_{x}^{1}-j_{x}^{2} \end{array}\right]$ is that two border nodes are not at the same point. Then, we can calculate *l* and *m* based on this. Now, the transformation matrix **T** is given by two border nodes *j*_1_ and *j*_2_ if we don’t consider the reflection transformation.

**Figure 3.**
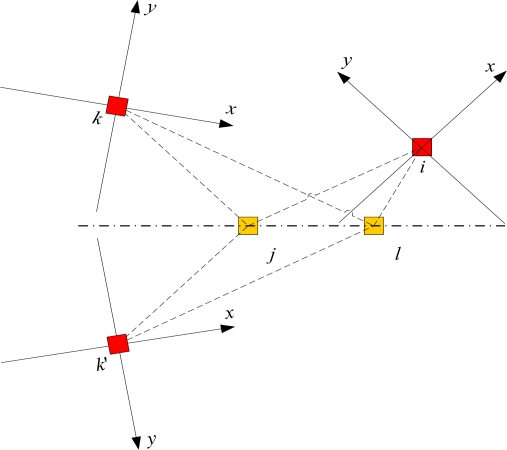
An example of the flip ambiguity problem in CSPA.

Of course, the deduction and conclusion mentioned above are not correct if we consider the reflection transformation. Suppose *xiy* and *xk’y* are local coordinate systems waiting to merge, nodes *j* and *l* are two border nodes, and *xky* and *xk’y* can be reflected across the line connecting nodes *j* and *l* while satisfying the distance constraint as shown in Figure 3. Since the coordinate systems *xiy* and *xky* are satisfied the same right-hand coordinate hypothesis, that is the positive *x* axis coincides with the positive *y* axis after rotating 90 degrees counter-clockwise about the origin, two local coordinate systems can coincide only using the translation and rotation transformation based on Equations 4 and 5. However, we need additional reflection transformation to obtain the coordinate systems *xk’y*. Thus, it can be seen that a major problem in CSPA based on distance measurements is the flip ambiguity, which introduces large errors in the location estimates considering the accumulated effect. More discussions on graph rigidity and network localization can be found in [33].

## 4. Theoretical Analysis

### 4.1. Analysis of Energy Consumption

Referring to the theoretical analysis method in reference [30], this section considers the energy consumption of Algorithm 1. The total power requirements include both the power required to transmit messages and the power required to receive (or process) messages.

When a sensor, say sensor *i*, meets the conditions of being a master node, it broadcasts message M_1_ and assigns cluster ID *i* to its neighboring sensors. Its neighboring sensors then transmit a signal M_2_ to their neighbor nodes with the distance information. During this clustering phase, (1+*N_i_*) transmissions and ( $N_{i}+\sum_{j\in C_{i}}N_{j}$) receptions are executed, where *N_i_* is the number of neighbor nodes of sensor *i*, and *C_i_* is the index set of neighboring sensors of sensor *i*. This procedure is applied to all master nodes and their slave nodes. Now let *N_T_* and *N_R_* denote the total number of transmissions and receptions for all master nodes, respectively. Hence:

(6) $$N_{T}=\sum_{i\in I}\left(1+N_{i}\right)$$

(7) $$N_{R}=\sum_{i\in I}\left(\sum_{j\in C_{i}}N_{j}+N_{i}\right)$$

where *I* is a index set of master nodes in the WSNs.

Suppose that the energy needed to transmit is *E_T_*, which depends on the node communication radius *R*, and the energy needed to receive is *E_R_*. From Equations 6 and 7, the total energy consumption, *E_total_*, for cluster formation in the wireless sensor network is:

(8) $$E_{\mathrm{total}}=N_{T}\cdot E_{T}+N_{R}\cdot E_{R}$$

We observe that the above analysis is suitable for any transmitting range. However, overly small transmission ranges may result in isolated clusters whereas overly large transmission ranges may result in a single cluster. Therefore, in order to optimize energy consumption and encourage linking between clusters, it is sensible to consider the minimum transmission power which will result in a fully connected network. This range assignment problem is investigated in [34], which shows that *R^d^n* ≈ *l^d^* ln*l* may be a good initial value for the search of optimized range assignment strategies to provide a strongly connected network and energy conservation. As usual, *n* is the number of sensors and *l* is the length of sides of a *d*-dimensional cube.

### 4.2. Parameter-setting Guidelines

We observe that there exist three main parameters in MSPA, including the multiplication factor *α*, node density *d* and node communication radius *R*. As has been noted, the multiplication factor *α* plays an important role in adjusting the number of master nodes and slave nodes in sensor networks and the degree of connectivity among master nodes. In practice, we can set parameter *α* depending on different motives and constraints. For the entire sensor network, we define the node density *d* as the number of nodes in the unit area. However, it is noted that *d* is defined as the average number of neighbor nodes in the range of each master node in [23]. This idea is less likely to be accepted because it is inconsistent with many widely accepted concepts, so we must study seriously the related conclusion from [23] and process it in different ways. As we all know, the node density *d* and node communication radius *R* are affected by capital cost, human resources, sensor performance and entire environment, *etc.* Under normal conditions, overly small node density and node communication radius may result in low localization success and bad algorithm performance, whereas overly large node density and node communication radius may result in a high sensor network cost and even be technically impossible for each sensor. Therefore, in order to optimize system performance and facilitate application, it is sensible to consider a universal parameter-setting guideline which will result in high feasibility and low cost of the entire sensor network.

According to previous analysis of the composition of MSPA, we can reach some conclusions as follows:

**Conclusion 1.** In the local coordinate system construction phase, except for the two slave nodes selected first, the coordinates of other slave nodes are determined by at least three neighbor nodes.
**Conclusion 2.** In the global coordinate system organization phase, the coordinates of border nodes are determined by at least four neighbor nodes when no master-slave relationships exist between the master nodes of two local coordinate systems.
**Conclusion 3.** In the global coordinate system organization phase, the coordinates of border nodes are determined by at least two neighbor nodes when there exists a master-slave relationship between the master nodes of two local coordinate systems.

Now, let us try to compute the approximate number of neighbor nodes of any node in MSPA.

**Theorem 1.** Let the nodes be distributed uniformly over a region of area *S*, the node density is *d* and the node communication radius is *R*. The number of neighbor nodes in the range of any master node is a random variable *X*. Then the probability function is:

(9) $$P(X=k)=\frac{1}{e^{\lambda }-1}•\frac{\lambda^{k+1}}{(k+1)!}$$

**Proof.** Let us consider such trials. In each trial, the node is deployed uniformly over a region and we call a trial successful when the node is deployed in a fixed area. For each node, thus, the probability of the trial to be successful (event 1) is $p=\frac{{\pi R}^{2}}{S}$ and the probability of failure (event 0) is *q* = 1 − *p*. If the trials occur independently, the entire *n* trials constitute a Bernoulli sequence. The distribution of the probability of nodes in a fixed area (random variable *Y*) is, therefore, a binomial distribution. The probability of exactly *k* nodes in a fixed area is then:

(10) $$P(Y=k)=C_{n}^{k}p^{k}q^{n-k}$$

When *n* is extremely large and *np* = *λ*, the Poisson Theorem gives us:

(11) $$P(Y=k)=\frac{e^{-\lambda }\lambda^{k}}{k!}$$

where $\lambda =\mathrm{np}=n\frac{{\pi R}^{2}}{S}=\frac{n}{S}•{\pi R}^{2}={d\pi R}^{2}$.

Given an extremely large *n* and *np* = *λ*, therefore, the distribution of probability of exactly *k* nodes in fixed area could be approximately a Poisson distribution.

Therefore, the probability function of random variable *X* can be obtained now as:

(12) $$P(X=k)=\frac{P(Y=k+1)}{P(Y\geq 1)}=\frac{P(Y=k+1)}{1-P(Y=0)}=\frac{\frac{e^{-\lambda }\lambda^{k+1}}{(k+1)!}}{1-e^{-\lambda }}=\frac{1}{e^{\lambda }-1}•\frac{\lambda^{k+1}}{(k+1)!}$$

and it is not difficult to compute that (for more details see Appendix B):

(13) $$E(X)=\frac{\lambda }{1-e^{-\lambda }}-1$$

(14) $$D(X)=\frac{\lambda }{1-e^{-\lambda }}(\lambda +1-\frac{\lambda }{1-e^{-\lambda }})$$

Iyengar has also noticed and investigated the similar problem [12], but he insisted that the probability that an arbitrary node has *k* neighbor nodes can be approximated by the Poisson distribution with parameter density *d*. But the truth is that random variable *X* is a conditional probability of random variable *Y* and the probability function of *X* is just similar to Poisson distribution in form. More importantly, the conclusions given above are obtained based on the hypothesis that *n* is extremely large and *np* = *λ*, which is also ignored by Iyengar.

## 5. Simulation Results

### 5.1. Parameter-Setting

**Figure 4.**
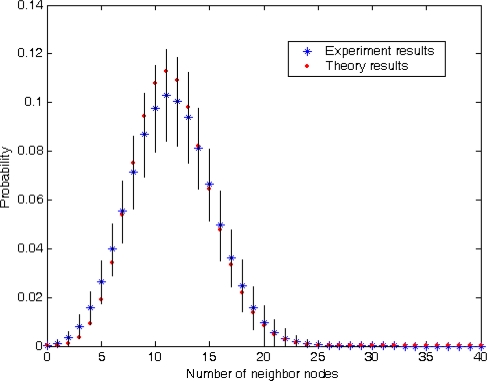
Probability distribution of the number of neighbor nodes.

The first set of experiments validates the parameter-setting guideline presented in Section 4.2. Assume that 400 sensors are uniformly distributed over a square region with side length 20 units and the node communication radius is 2 units. There are very different communication ranges in different sensors with different hardware and abundant functions [35]. By taking into account the complexity of the real world, we use of the word unit to represent different length such as 0.5(*m*), 1(*m*) or 10(*m*). Figure 4 gives a comparison between theory analytical results and results of 1,000 repeated experiments with the same parameter. In Figure 4, the length of each black line is two times the standard deviation of these independent experiments. Obviously, as shown in Figure 4, the error of experiments is still promising and the analytical results show a good match with the experimental results. Table 1 presents the results on mean value and standard deviation of the number of neighbor nodes. It shows that the mean value of the theoretical results almost coincides with the experimental results, and the error of standard deviation between the theoretical and experimental results is just 6%. All this fully confirms the validity of the theoretical analysis of the random variable *X* as shown in Equations 12–14.

**Figure 5.**
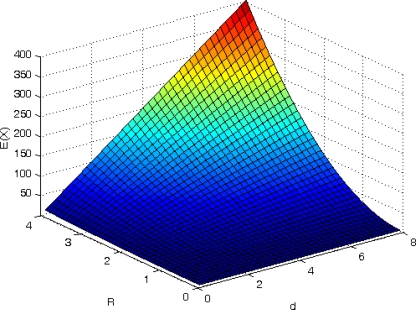
*E*(*X*) with respect to *d* and *R*.

Since the theory derivation process doesn’t involve the algorithm, we may have reason to believe that Theorem 1 is of universal significance among all relative localization algorithms. What is more important to us here is that it provides a parameter-setting guideline for node density and node communication radius and an answer to which requirement must be met before the GPS-free localization system becomes applicable. Figure 5 shows the mean value *E*(*X*) with respect to node density *d* and node communication radius *R*. We observe that the average neighbor nodes in the range of each master node increases and the coordinates of more nodes in the sensor network can be determined by MSPA as the *d* and *R* increases, which coincides with our experience and general knowledge. We also find out that the mean value reveal itself approximates an exponential increase for a large enough *d* or *R*. The reason is that, as we can see from Equation 13, when *d*(*R*) is constant and *R*(*d*) is large enough, $E(X)=\frac{\lambda }{1-e^{-\lambda }}-1=\frac{{d\pi R}^{2}}{1-e^{{-d\pi R}^{2}}}-1\propto e^{{d\pi R}^{2}}$. Therefore, the mean value follows approximate exponential increase with *d*(*R*^2^).

**Figure 6.**
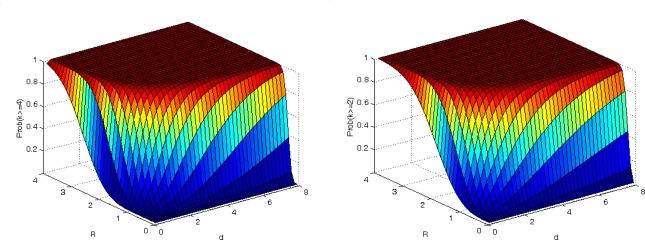
*P*(*X* ≥ *k*) with respect to *d* and *R*. (a) *k* = 4. (b) *k* = 2.

As we can see from conclusions 1–3, the fundamental condition in an efficient implementation of MSPA is that the number of neighbor nodes in the range of any node is 4. The smallest number of neighbor nodes is 2, and otherwise it is impossible in theory to determine the coordinates of all nodes in the network. In Figure 6 we show the probability that a node has 4 or more and 2 or more neighbor nodes as a function of *d* and *R*. We note that the probability increases as *d* and *R* increase. Furthermore, the contour line at the same probability is a part of approximate ellipse, in which major axis is *d* and minor axis is *R*. The probability undergoes a rapid change during a certain interval of major axis and minor axis and then tends to steady-state. We gain enlightenment from this that it is good choice for *d* and *R* to maintain the probability in steady state and avoid changing intervals in a real world.

Since the square region in a two-dimensional plane is fixed, the larger the node density, the more nodes as deployed and thus the more the capital cost and human resources are. A noticeable discrepancy between high technical requirements and limited power and volume of each sensor exists, with the implication that some moderate communication radius may be a good choice. Under comprehensive consideration of resource consumption and algorithm performance, in the following experiments, we assume that 400 sensors are uniformly distributed over a square region with side length 20 units and node communication radius is 2 units. The parameters of the Poisson distribution for this scenario are $\lambda =\mathrm{np}=400\times \frac{\pi \times 2^{2}}{20\times 20}=4\pi$, *E*(*X*) = 11.57, *P*(*X*≥4) = 99.49%, *P*(*X* ≥ 2) = 99.97%, which fits the requirements of the MSPA algorithm.

### 5.2. Typical Result

**Figure 7.**
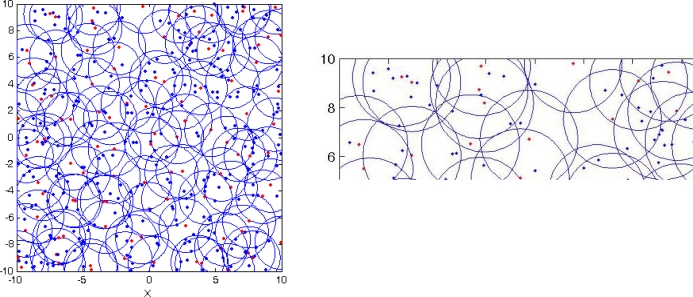
Sensors distribution and communication range of sensor nodes. (a) Global map. (b) Partial enlargement map.

**Figure 8.**
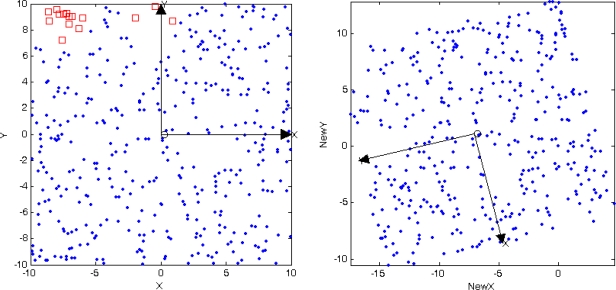
Localization result of MSPA. (a) Global localization result (b) Optimal localization result.

The second set of experiments in Figures 7 and 8 show the typical result of MSPA. Figure 7 depicts the typical sensor distribution of the WSNs and the communication range of sensor nodes. The red dots denote master nodes, blue dots denote slave nodes and border nodes, and the blue circles denote the communication domain of each master node. It can be seen from Figure 7(a) that some master nodes are deployed within the communication range of other master nodes. So, from another perspective, these master nodes with master-slave relationships are part of the border nodes. This is due to the fact that multiplication factor *α* plays an important role in the adjusting the degree of connectivity among master nodes.

Figure 8 depicts the typical runs of the proposed MSPA and Figure 8(b) also shows that the optimal localization result. In Figure 8, the blue and red dots denote successful localization sensor nodes and lost localization sensor nodes respectively, and *XOY* is the absolute coordinate system. Compared with absolute coordinate system *XOY*, the relative coordinate system undergoes translation, rotation and reflection transformations, as shown in Figure 8(b). As expected, the distance and orientation between sensors do not change at all compared with the absolute coordinate system. Then, these characteristics of relative coordinate system in WSNs are essential for any applications.

In general, the coordinate information of most nodes in sensor network can de determined by using MSPA, as seen in Figure 8(a). On the other hand, since the sensors are randomly deployed in a square area, it is always possible for some sensors to be isolated from most of the other sensor nodes. The result is we may not obtain any knowledge about their coordinates no matter how we set the parameters. For example, as shown in Figure 7(b) and 8(a), all nodes in the sensor work area are localized in three different local coordinate systems, the largest of which is defined as the global relative coordinate system. Clearly, the other two coordinate systems don’t meet the requirements of merging with the global coordinate system, that is the three border nodes, and are isolated.

### 5.3. Effects of Different Parameters

**Figure 9.**
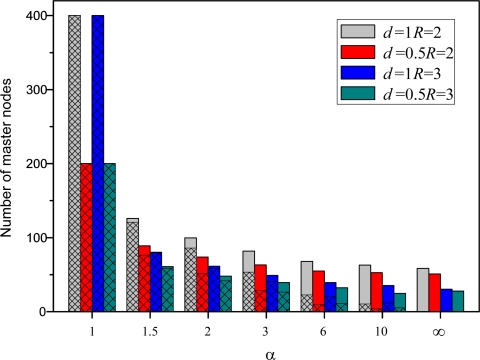
Average number of master nodes with respect to different parameters.

The third set of experiments examines the effect of different parameters, such as the multiplication factor *α*, node density *d* and node communication radius *R*, on the algorithm’s performance. In this paper, the four useful evaluation criteria we used are the number of master nodes including master-slave relationships or not, the number of local coordinate systems, the success rate of localization and convergence time. The number of master nodes gives expression to the results of nodes categories or clustering. The number of local coordinate systems and success rate of localization are a performance measure that can be used to evaluate the accuracy of GPS-free localization algorithms. The last metric tells us the length of time required by a sensor network to perform the algorithm when it generates a global coordinate system. The average number in each case is the simple mean of the results of 200 typical runs. Figure 9 shows the variation of the average number of master nodes. In the figure, grid lines and blank space denote master nodes with master-slave relationships or not, respectively, and different colors denote different parameter-settings.

The graph suggests the number of master nodes decreases and the number of master nodes without master-slave relationships increase as the multiplication factor *α* increases. This is due to the fact that a large number of waiting timers would be deleted and the corresponding nodes have a great chance to become master nodes when the multiplication factor *α* is large. An additional result that is perhaps interesting is that the distance between adjacent master nodes is increasing and more and more master nodes cannot communicate directly. It is specially noted that, when *α* = 1, all nodes in the sensor network are master nodes as we mentioned above. Since there have no concept of slave nodes, the so-called problem of master-slave relationship between master nodes is meaningless, even though all nodes are denoted by grid lines in Figure 9. On the other hand, when *α* = ∞, each master node cannot be connected with any other master nodes, that is to say, all master nodes are not border nodes. In this special case, although the number of master nodes decreases, the requirements of number and distribution of border nodes is higher than other case in order to merge the two adjacent coordinate systems.

We can also observe from Figure 9 that the average number of master nodes decrease as node density decrease and node communication radius increase. Since larger transmission power allows larger communication coverage, a master sensor has more neighbor sensors, which reduces the number of master sensors in the network. As one would expect, the ratio of master sensors to all sensors in the network increase and the number of master node decrease as node density decrease.

**Figure 10.**
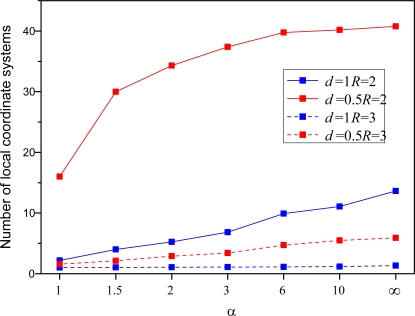
Average number of local coordinate systems with respect to different parameters.

**Figure 11.**
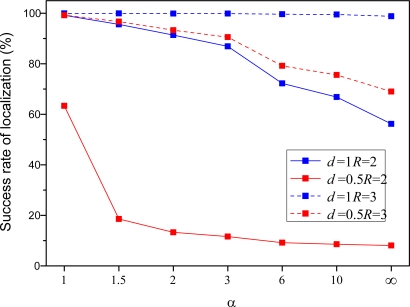
Average success rate of localization with respect to different parameters.

Figures 10 and 11 show the average number of local coordinate systems and localization success rate with the respect of different parameters, where localization success rate can be expressed as a percentage by the number of sensors in the largest local coordinate system divided by the total number of sensors in the WSNs. The number of local coordinate systems increases and localization success rate decreases as multiplication factor *α* increases. As shown in Figure 9, the number of master nodes also decreases as the multiplication factor *α* increases. The result is that the accuracy of the proposed algorithm becomes worse and worse with larger distances between adjacent master sensors.

What is more, we can also conclude from Figures 10 and 11 that large node density and node communication radius can lead to promising localization performance and comparatively speaking, node density exerts more pressure upon the algorithm. More precisely, it is found that the localization success rate is only 10–20%, whereas it is up 60%, increasing four-fold or five-fold, when the node density increases from 0.5 to 1. Clearly, it is crucial to improve each sensor’s performance and enlarge the communication radius under the premise that the transmission power, energy and volume don’t change dramatically; otherwise, the localization accuracy of algorithm would be reduced significantly. Thus, it is not a good idea to deploy a large number of sensors with bad performance.

**Figure 12.**
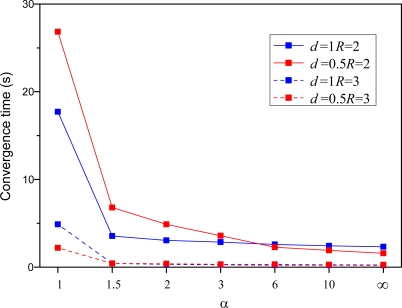
Average convergence time with respect to different parameters.

In Figure 12, we have made a similar comparison on convergence time. Figure 12 shows that convergence time decrease as multiplication factor *α* increase. When multiplication factor *α* is larger than 1.5, especially than 3, the convergence time tend to constant with minor variety. It can be seen from Figures 9–12 above that little multiplication factor is a good solutions to improve the localization accuracy with less time cost. Therefore, we will take use of *α* = 2 in the following simulations considering algorithm performance and implement time. In addition, compared with node density, node communication radius play more important role to algorithm performance, which also indicates the important of each sensor performance in WSNs.

### 5.4. Performance Comparison with Other Algorithms

The final set of experiments compares the performance of the proposed algorithm with that of other relative algorithms in terms of the number of master nodes, accuracy and convergence time like in Section 5.3. As we all know, since the coordinates knowledge of two border nodes are used in organizing the local coordinate systems, the main problem of CSPA is the flip ambiguity in sensor networks, as shown in Figure 3. For the sake of avoiding flip ambiguity in theory in this paper, the CSPA [16] is extended to the CSPA+ without changing the core idea and structure of algorithm, whereas its merging of local coordinate systems is dependent on the coordinates of three border nodes. All algorithms are programmed by MATLAB 7.0 language and experimental data are analyzed and processed by SPSS 14.0. The running environment is a Pentium IV 2.4 GHz PC and RAM is 512 MB. We make 200 independent experiments, all with the same parameter-setting, and then these experiments results are averaged together.

**Figure 13.**
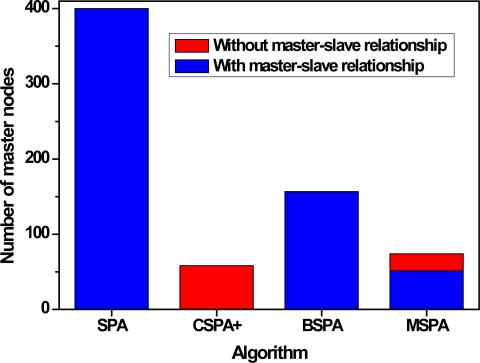
Average number of master nodes with respect to different algorithms.

Figure 13 shows the average number of master nodes after constructing local coordinate systems with different algorithms. Since the CSPA+, BSPA[23] and our MSPA algorithms are all based on clusters, the number of master nodes decrease markedly compared with SPA [14,15]. Within all the algorithms, the number of master nodes obtained by the CSPA+ algorithm is the least and specially all sensors are not in the range of other sensors as we expect. On the other hand, the number of master nodes obtained by BSPA is comparably larger and almost of all master nodes have a master-slave relationship with another node. In theory, all sensors in a sensor network are connected with other sensors, whereas few sensors are isolates and cannot communicate with all other sensors for uniform distribution in a practical work like this paper. The number of master nodes obtained by our MSPA would fall in between them; furthermore we can control the number and relationships among master nodes, which is flexible and suitable for engineering applications. It is necessary to note that all nodes in the sensor network are master nodes obtained by SPA as we mentioned above. Since there have no concept of slave node, the so-called problem of master-slave relationship between master nodes is meaningless, no matter how it is denoted in Figure 13.

**Figure 14.**
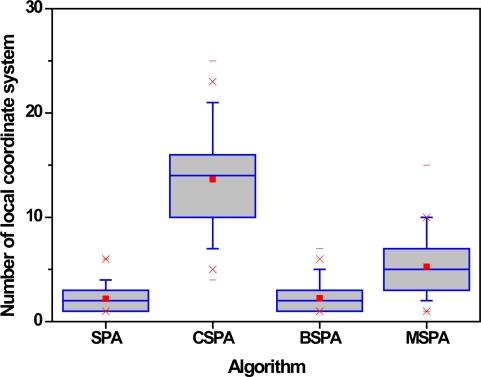
Number of local coordinate system with respect to different algorithms.

**Figure 15.**
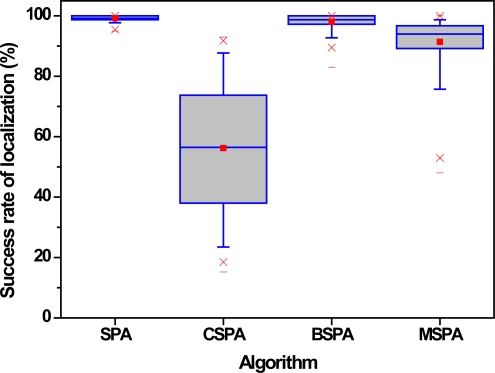
Success rate of localization with respect to different algorithms.

Figures 14 and 15 show the number of local coordinate systems and localization success rate with respect to the different algorithms. Experimental results from Figures 14 and 15 show, first, that the performance of SPA and BSPA is very similar. It is also apparent that they maintain the highest level of accuracy and stability. The reason is that the sensors in network connected with all master nodes always can communicate directly, which is propitious to merge them into a global coordinate system. On the other hand, for the similar reason, the performance of CSPA+ is so low that it cannot be used in some situations with high accuracy and stability requirements. Clearly, the performance of MSPA algorithm is also fall in between them. However, its predominance is adjustable by different parameters in different situations.

**Figure 16.**
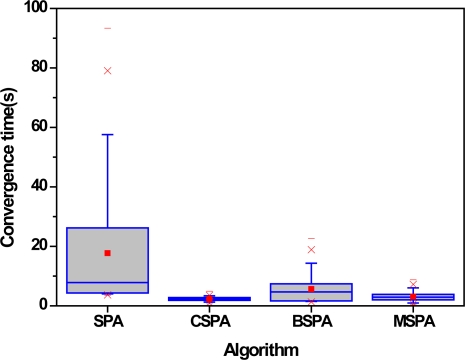
Convergence time with the respect to different algorithms.

Figure 16 shows the convergence time with the respect to the different algorithms. It can be seen that the temporal efficiency and stability of SPA is very bad as its convergence time is the longest among all algorithms considered. The most extreme example provided in our present study is 93 seconds. In addition, the mean and median of convergence time required by BSPA are 5.6 seconds and 4.8 seconds, respectively. On the other hand, the convergence time of CSPA+ and MSPA algorithm is the shortest. The reason is that they can reduce the merging operation for the least number of master sensor nodes in WSNs, as shown in Figure 13.

## 6. Conclusions

This paper has presented a GPS-free localization scheme for wireless sensor networks, which does not rely on any absolute position reference such as GPS or fixed anchor nodes. The proposed MSPA algorithm combines a standardized clustering based approach to construct local coordinate systems and a transformation matrix based approach to merge them into a global coordinate system, which can complete solve the flip ambiguity problem in WSNs. The effectiveness of the proposed algorithm has been demonstrated through simulation. Also, we have developed a set of parameter-setting guidelines for the proposed algorithm based on a probability model, and investigated the energy requirements.

Despite the results obtained in this paper give positive answers to a number of the problems that motivated our study, there are still many unsolved problem in the area. Since the sensors are equipped with limited energy supplies, one of the primary goals is to reduce the overall energy consumption of the network, thus increasing its lifetime. As part of our continuing research, we plan to discuss more technical issues about the energy consumption problem to verify the performance of the proposed scheme. Additionally, we are interested in testing and improving the performance of our algorithm under distance measurement errors so that it will be more feasible in real world applications. Finally, we envision extending it for three dimensional model and mobile wireless sensor networks.

## Figures and Tables

**Table 1.** Comparisons of mean value and standard deviation between theoretical and experimental results.

	**Mean value**	**Standard deviation**
**Theoretical results**	0.02439024390119	0.03728029674933
**Experimental results**	0.02439024390244	0.03513608621224

**Figure 17.**
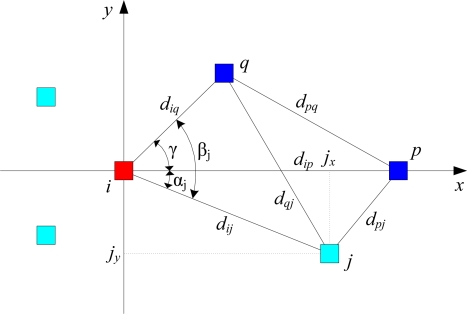
An example illustrating the way to obtain the position of node *j* in the coordinate system of node *i*.

## Appendix A

In this section we show how each master node builds its local coordinate system. The master node becomes the center of its own coordinate system with the position (0, 0) and the positions of its slave nodes are computed accordingly.

The node *j* is called a one-hop neighbor of node *i* if nodes *i* and *j* can communicate directly (in one hop). Let **N** be the set of all the nodes in the network. We define ∀ *i* ∈ **N**, a set of nodes **K_i_** such that ∀ *j* ∈ **K_i_**, *j* ≠ *i*, *j* is a one hop neighbor of *i*. We call **K_i_** the set of one-hop neighbors of node *i*. Denote the distance between any two nodes, *p* and *q* by *d_pq_*. A coordinate system can then be established, if there exists two nodes *p*, *q* ∈ **K_i_** such that *d_pq_* is known at node *i*. With the master node *i* being the origin of the coordinate system, either node *p* (or node *q*) can be defined to lie on the positive *x* axis. Node *q* (or node *p*) is now assumed to have a positive *y* component to define the *y* axis and the coordinates of the nodes *i*, *p* and *q* are given by:

(15) $$\begin{array}{c} i_{x}=0;\;\;i_{y}=0;\\ p_{x}=d_{\mathrm{ip}};p_{y}=0;\\ q_{x}=d_{\mathrm{iq}}\;\text{cos}\;\gamma ;\;q_{y}=d_{\mathrm{iq}}\;\text{sin}\;\gamma \end{array}$$

where *γ* is the angle ∠ (*p*,*i*,*q*) in the triangle ▵ (*p*,*i*,*q*) and it is obtained by using a cosine rules for triangles:

(16) $$\gamma =\text{arccos}\frac{d_{\mathrm{iq}}^{2}+d_{\mathrm{ip}}^{2}-d_{\mathrm{pq}}^{2}}{{2d}_{\mathrm{iq}}d_{\mathrm{ip}}}$$

The positions of other node *j*, *j* ∈ **K_i_**, *j* ≠ *p*,*q*, for which *d_ij_*, *d_qj_* and *d_pj_* are known, are computed by triangulation. Therefore, we obtain:

(17) $$j_{x}=d_{\mathrm{ij}}\;\text{cos}\;\alpha_{j}$$

(18) $$j_{y}=\left\{ \begin{array}{l} d_{\mathrm{ij}}\;\text{sin}\;\alpha_{j}\;\;\text{if}\;\beta_{j}=|\alpha_{j}-\gamma |\\ -d_{\mathrm{ij}}\;\text{sin}\;\alpha_{j}\;\text{else} \end{array}\right.$$

where, *α_j_* is the angle ∠ (*p*,*i*,*j*) in the triangle ▵ (*p*,*i*,*j*) and *β_j_* is the angle ∠ (*j*,*i*,*q*) in the triangle ▵ (*j*,*i*,*q*). In practice, *β_j_* will never be exactly equal to |*α_i_* – *γ*| due to the errors in distance measurements. The purpose of this exercise is to find on which side of the *x* axis node *j* is located and some difference between these two values that needs to be tolerated. Then, we obtain the values of *α_j_* and *β_j_* by using the cosine rule:

(19) $$\alpha_{j}=\text{arccos}\frac{d_{\mathrm{ij}}^{2}+d_{\mathrm{ip}}^{2}-d_{\mathrm{jp}}^{2}}{{2d}_{\mathrm{ij}}\;d_{\mathrm{ip}}}$$

(20) $$\beta_{j}=\text{arccos}\frac{d_{\mathrm{ij}}^{2}+d_{\mathrm{iq}}^{2}-d_{\mathrm{jq}}^{2}}{{2d}_{\mathrm{ij}}\;d_{\mathrm{iq}}}$$

Figure 17 shows an example of the computation of node *j*. The positions of the nodes *k* ∈ **K_i_**, *k* ≠ *p*,*q*, and *k* ∉ **K_p_**, *k* ∉ **K_q_**, can be computed by using the positions of the node *i* and at least two other nodes for which the positions are already obtained, if the distance from the node *k* to these nodes is known.

## Appendix B

As noted above, the number of neighbor nodes in the range of any master node is random variable *X*. The probability function is:

(21) $$P(X=k)=\frac{1}{e^{\lambda }-1}•\frac{\lambda^{k+1}}{(k+1)!}$$

where $\lambda =\mathrm{np}=n\frac{{\pi R}^{2}}{S}=\frac{n}{S}•{\pi R}^{2}={d\pi R}^{2}$.

Obviously:

(22) P(X=k)≥0, ∀k=0,1,2, …

and:

(23) $$\begin{array}{l} \sum_{k=0}^{\infty }P(X=k)=\frac{1}{e^{\lambda }-1}•\sum_{k=0}^{\infty }\frac{\lambda^{k+1}}{(k+1)!}\\ =\frac{1}{e^{\lambda }-1}•\sum_{k=1}^{\infty }\frac{\lambda^{k+1}}{k!}\\ =\frac{1}{e^{\lambda }-1}•(\sum_{k=0}^{\infty }\frac{\lambda^{k}}{k!}-1)\\ =\frac{1}{e^{\lambda }-1}•(e^{\lambda }-1)\\ =1 \end{array}$$

Hence, Equation 21 ultimately meets the definition of probability function of discrete random variable.

The mean value:

(24) $$\begin{array}{l} E(X)=\frac{1}{e^{\lambda }-1}•\sum_{k=0}^{\infty }k\frac{\lambda^{k+1}}{(k+1)!}\\ =\frac{1}{e^{\lambda }-1}•[\sum_{k=0}^{\infty }(k+1)\frac{\lambda^{k+1}}{(k+1)!}-\sum_{k=0}^{\infty }\frac{\lambda^{k+1}}{(k+1)!}]\\ =\frac{1}{e^{\lambda }-1}•[\sum_{k=0}^{\infty }\frac{\lambda^{k+1}}{k!}-\sum_{k=1}^{\infty }\frac{\lambda^{k}}{k!}]\\ =\frac{1}{e^{\lambda }-1}•[\lambda \sum_{k=0}^{\infty }\frac{\lambda^{k}}{k!}-(\sum_{k=0}^{\infty }\frac{\lambda^{k}}{k!}-1)]\\ =\frac{1}{e^{\lambda }-1}•[\lambda e^{\lambda }-(e^{\lambda }-1)]\\ =\frac{\lambda }{1-e^{-\lambda }}-1 \end{array}$$

Notice that:

(25) $$\begin{array}{l} E(X^{2})=\frac{1}{e^{\lambda }-1}•\sum_{k=0}^{\infty }k^{2}\frac{\lambda^{k+1}}{(k+1)!}\\ =\frac{1}{e^{\lambda }-1}•[\sum_{k=0}^{\infty }k(k+1)\frac{\lambda^{k+1}}{(k+1)!}-\sum_{k=0}^{\infty }k\frac{\lambda^{k+1}}{(k+1)!}]\\ =\frac{1}{e^{\lambda }-1}•[0+\sum_{k=1}^{\infty }\frac{\lambda^{k+1}}{(k-1)!}]-E(X)\\ =\frac{1}{e^{\lambda }-1}•\sum_{k=0}^{\infty }\frac{\lambda^{k+2}}{k!}-E(X)\\ =\frac{\lambda^{2}}{e^{\lambda }-1}•\sum_{k=0}^{\infty }\frac{\lambda^{k}}{k!}-E(X)\\ =\frac{\lambda^{2}}{e^{\lambda }-1}•e^{\lambda }-E(X) \end{array}$$

Therefore, the variance is:

(26) $$\begin{array}{l} D(X)=E(X^{2})-E^{2}(X)\\ =\frac{\lambda^{2}}{e^{\lambda }-1}•e^{\lambda }-(\frac{\lambda }{1-e^{-\lambda }}-1)-{\left(\frac{\lambda }{1-e^{-\lambda }}-1\right)}^{2}\\ =\frac{\lambda^{2}}{e^{\lambda }-1}•e^{\lambda }-\frac{\lambda }{1-e^{-\lambda }}+1-\frac{\lambda^{2}}{{\left(1-e^{-\lambda }\right)}^{2}}+\frac{2\lambda }{1-e^{-\lambda }}-1\\ =\frac{\lambda }{1-e^{-\lambda }}(\lambda +1-\frac{\lambda }{1-e^{-\lambda }}) \end{array}$$
